# Differential expression of miRNAs in esophageal cancer tissue

**DOI:** 10.3892/ol.2013.1251

**Published:** 2013-03-12

**Authors:** SHANG-GUO LIU, XIU-GUANG QIN, BAO-SHENG ZHAO, BO QI, WEN-JIAN YAO, TIAN-YUN WANG, HAN-CHEN LI, XIANG-NAN WU

**Affiliations:** 1 Department of Thoracic Surgery, The First Affiliated Hospital of Xinxiang Medical University, Weihui 453100;; 2 Department of Biochemistry and Molecular Biology, Xinxiang Medical University, Henan, Xinxiang 453003, P.R. China

**Keywords:** microRNA, expression profile, esophageal cancer, gene chip, real-time polymerase chain reaction

## Abstract

The aim of this study was to explore the association of specific microRNAs (miRNAs) with the development of esophageal cancer (EC) and to identify new molecular markers for EC by analyzing the expression profiles of miRNAs in EC tissues. The expression profiles of miRNAs in paired EC and paracancerous normal tissues were detected and bioinformatically analyzed using miRNA assays. The outcomes were validated using real-time polymerase chain reaction. The miRNA assays revealed a total of 60 differentially expressed miRNAs in the EC tissues compared with those in the paracancerous normal tissues. Among them, 51 had doubled or more than doubled their expression levels and 9 had halved their expression levels. The most markedly upregulated miRNAs were hsa-miR-15a, hsa-miR-28-3p, hsa-miR-31, hsa-miR-99b, hsa-miR-101, hsa-miR-130a, hsa-miR-143, hsa-miR-196b, hsa-miR-200a, hsa-miR-210, hsa-miR-452 and hsa-miR-27a, whereas the most markedly downregulated miRNAs included hsa-miR-30b, hsa-miR-223, hsa-miR-454, hsa-miR-486, hsa-miR-574-3p and hsa-miR-126. Specific miRNA expression profiles exist in EC tissues and may serve as novel EC molecular markers.

## Introduction

Esophageal cancer (EC) is one of the six most common malignancies worldwide, with a higher incidence in males than females (1). In China, EC has a mortality rate of 17.38/100,000 and ranks fourth among malignancies following gastric cancer, lung cancer and hepatocarcinoma. EC has an insidious onset; therefore, most patients have lapsed into advanced stage disease by the time of final diagnosis. The first-line treatment method for EC is currently a combination of surgery, chemotherapy and radiotherapy. However, this method only achieves a five-year survival rate of 5–10%. Within one year of the final diagnosis, 75% of patients succumb to EC (2). Therefore, identifying biological markers for EC to improve the early diagnosis rate and developing gene therapies is important for reducing the mortality rate of EC.

microRNAs (miRNAs) are a class of conservative single-stranded non-coding RNAs, composed of 17–25 ribonucleotides (3). Previous studies have shown that miRNA is involved in numerous physiological processes of cell regulation, including differentiation, proliferation, apoptosis and metabolism, and is also important in the development of cancer. A number of miRNAs function as oncogenes, while others may function as anti-oncogenes. Therefore, miRNAs have the potential to become new biological markers for EC and to be applied in the diagnosis, prognosis and targeted treatment of EC (4).

Tumors are subject to complicated medical conditions. The majority of tumors develop concurrently with the overexpression of oncogenes and/or the loss of anti-oncogenic expression. Almost half of human miRNAs are localized in tumor-associated genetic or fragile regions, including amplification, loss of heterozygosity, fragile and oncogene or anti-oncogene breakpoint regions (5). This suggests that miRNA has a role in the development of tumors. Studies have demonstrated that numerous miRNAs are directly involved in the initiation and development of EC and that their expression is correlated with the diagnosis, staging, progression and prognosis of EC (6–10).

Although studies have shown that miRNAs are important in the initiation, development, invasion and metastasis of tumors, their underlying mechanisms of action remain unknown. Whether the abnormal expression of miRNA is subject to the premise of tumorigenesis or its consequence, the mechanisms via which tissue-specific miRNAs act on different genes and the various factors which may regulate miRNA expression remain to be explored.

Binzhou and adjacent areas, including Huixian and Anyang in the Henan province of China, have a high incidence and mortality rate for EC. At present, EC remains the major cause of tumor-associated mortality in these areas (11). Since the molecular mechanisms underlying the initiation and development of EC remain unclear, a sensitive and specific molecular marker for the early diagnosis of EC has not yet been identified. This may be one of the major reasons for the high mortality rate and poor prognosis of this condition (12). In the current study, miRNA spectra of three pairs of EC and paracancerous normal tissues were detected. The differentially expressed miRNAs were analyzed. The aim of this study was to provide a basis for further exploration of the mechanisms underlying the development of EC, in addition to determining molecular markers associated with EC for its early diagnosis and treatment.

## Materials and methods

### Clinical data

Three EC specimens from the First Affiliated Hospital of Xinxiang Medical University were collected after first resection of the primary lesions. Fresh normal mucous membranes of the esophagus at the same pathological stage, 8 cm from the border of the tumor tissues were collected for the control samples. All resected tissue samples were stored at −80°C after freezing with liquid nitrogen. This study was conducted in accordance with the Declaration of Helsinki and with approval from the Ethics Committee of Xinxiang Medical University. Written informed consent was obtained from all participants.

### Sample treatment and RNA extraction

Total RNA was extracted from the oncocytes in the sections using the TRIzol one-step method according to the manufacturer’s instructions (Invitrogen, Carlsbad, CA, USA). Total RNA was collected according to the manufacturer’s instructions in the Recover All Total Nucleic Acid Isolation kit TRIzol reagent (Applied Biosystems, Carlsbad, CA, USA). Light absorption values were read at 230, 260 and 280 nm using spectrophotometry to determine purity and density. Formaldehyde-agarose gel electrophoresis was performed for the 28S:18S ratio to determine the purity and integrity of the total RNA sample.

### Real-time quantitative reverse transcription polymerase chain reaction (RT-qPCR)

miRNAs were quantitated using RT-qPCR. The Megaplex Pool reverse transcription system (Applied Biosystems) was prepared with a total volume of 4.5 *μ*l, containing Megaplex™ RT primers (10X) at 0.8 *μ*l, dNTPs with dTTP (100 mM) at 0.2 *μ*l, MultiScribe™ reverse transcriptase (50 U/*μ*l; Applied Biosystems) at 1.5 *μ*l, 10X RT buffer at 0.8 *μ*l, MgCl_2_ (25 mM) at 0.9 *μ*l, RNase inhibitor (20 U/*μ*l) at 0.1 *μ*l and nuclease-free water at 0.2 *μ*l. The solution was reverse transcribed six times and then centrifuged. Total RNA at 3 *μ*l (1–350 ng) was added into the reaction tube, reverse transcribed six times, centrifuged and placed on ice for 5 min. The PCR conditions consisted of 40 cycles of 16°C for 2 min, 42°C for 1 min and 50°C for 1 sec, followed by 85°C for 5 min and a termination step at 4°C. The reverse transcription products were placed on ice.

The pre-amplification PCR system was prepared with a total volume of 25 *μ*l, containing TaqMan^®^ PreAmp Master Mix (2X; Applied Biosystems) at 12.5 *μ*l, Megaplex™ PreAmp primers (10X) at 2.5 *μ*l, RT product at 2.5 *μ*l and nuclease-free water at 7.5 *μ*l. The solution was reverse transcribed six times, centrifuged and then placed on ice for 5 min. The PCR conditions consisted of 95°C for 10 min*,* 12 cycles of 55°C for 2 min, 72°C for 2 min, 95°C for 15 sec and 60°C for 4 min, followed by 99.9°C for 10 min and a termination step at 4°C.

TE (0.1X; pH 8.0) at 75 *μ*l was added into the pre-amplification tube, reverse transcribed six times and then stored at −20°C. TaqMan universal PCR Master mix was swirled to blend.

Regents with a total volume of 900 *μ*l, including TaqMan^®^ universal PCR Master mix, No AmpErase^®^ UNG (2X) at 450 *μ*l, diluted PreAmp product at 9 *μ*l and nuclease-free water at 441 *μ*l, were added into a 1.5-ml centrifuge tube, reverse transcribed six times and centrifuged.

The PCR mix at 100 *μ*l was applied to each sampling point and then twice centrifuged at 1,200 rpm (1 min each time). The reaction conditions consisted of 94.5°C for 10 min, 40 cycles of 97°C for 30 sec and 59.7°C for 1 min.

### Statistical analysis

The U6 snRNA housekeeping gene was used as an internal reference. The relative quantitative method was used. Gene expression was quantitated based on the following formula: F=2^−ΔΔct^ where ΔΔct = (ct mean of the target gene in the test sample - that of the housekeeping gene in the test sample) - (ct mean of the target gene in the control sample - that of the housekeeping gene in the control sample). A higher F-value indicates higher expression levels.

## Results

As shown in Table I, compared with the normal tissues, tumor tissues exhibited 60 differentially expressed miRNAs among the total 770. From the differentially expressed miRNAs, 51 were upregulated and 9 were downregulated. A number of miRNAs were upregulated >20 times, including hsa-miR-15a, hsa-miR-28-3p, hsa-miR-31, hsa-miR-99b, hsa-miR-101, hsa-miR-130a, hsa-miR-143, hsa-miR-196b, hsa-miR-200a, hsa-miR-210, hsa-miR-452 and hsa-miR-27a, whereas those which were downregulated >5 times included hsa-miR-30b, hsa-miR-223, hsa-miR-454, hsa-miR-486, hsa-miR-574-3p and hsa-miR-126 (Table I).

## Discussion

EC is a common malignancy and China is the country in which the highest morbidity and mortality rates for EC occur. The development of EC involves multiple factors, numerous genes and several stages. As most EC cases have progressed to advanced stage disease by the time of final diagnosis, EC patients always have a poor prognosis, with a five-year survival rate between 5 and 20%. Previous studies have revealed that the majority of ECs develop concurrently with the overexpression of oncogenes and/or the loss of anti-oncogenic expression (13,14). Almost half of miRNAs are localized at the fragile sites or in associated genetic regions of these genes, where they exert post-transcriptional control over the genes. Additionally, studies have shown that miRNAs tend to be expressed abnormally in tumor tissues (15,16). miRNAs function as tumor-inhibiting or cancerogenic factors in the development of tumors and also have extensive application value for diagnosing and predicting the prognosis of tumors.

In this study, miRNA expression in the EC and normal tissues was determined using miRNA chip technology. EC-associated miRNA spectra were obtained. Significantly upregulated miRNAs in the EC tissues included hsa-miR-15a, hsa-miR-28-3p, hsa-miR-31, hsa-miR-99b, hsa-miR-101, hsa-miR-130a, hsa-miR-143, hsa-miR-196b, hsa-miR-200a, hsa-miR-210, hsa-miR-452 and hsa-miR-27a. miRNAs which were downregulated >5 times included hsa-miR-30b, hsa-miR-223, hsa-miR-454, hsa-miR-486, hsa-miR-574-3p and hsa-miR-126. The 31 miRNA spectra obtained from the EC tissues were further analyzed using microassay technology. Results showed that 60 miRNAs exhibited abnormal expression. Guo *et al*(9) have shown that three miRNAs (hsa-miR-25, hsa-miR-424 and hsa-miR-151) are upregulated and four miRNAs (hsa-miR-100, hsa-miR-99a, hsa-miR-29c and mmu-miR-140) are reduced in cancer tissue compared with normal tissue. The results of this study showed that hsa-miR-25 was upregulated one and a half times in the tumor tissues, which is consistent with the results of Guo *et al*. However, this study also showed that hsa-miR-100, hsa-miR-99a and hsa-miR-29c were markedly upregulated in the tumor tissues, which contradicts the results of Guo *et al*. This disagreement is possibly ascribed to the small sample size, source of materials and EC stage in this study. Zhang *et al*(17) concluded, based on the comparison between 209 EC and 140 control samples, that seven serum miRNAs (miR-10a, miR-22, miR-100, miR-148b, miR-223, miR-133a and miR-127-3p) were significantly upregulated in the sera of esophageal squamous cell carcinoma patients compared with control individuals. This study also showed that the EC tissues exhibited upregulated miR-100 and miR-133a compared with normal tissues.

In conclusion, a total of 60 miRNAs were abnormally expressed in EC tissues compared with those in normal tissues. 51 miRNA fragments were upregulated ≥2 times and 9 were downregulated to less than half. However, considering the small number of samples employed in this study, our results require confirmation using a larger sample size.

## Figures and Tables

**Table I:** miRNA expression in esophageal cancer and normal tissue.

miRNA	Esophageal cancer tissue	Normal tissue	Esophageal cancer tissue/normal tissue regulation	Fold
hsa-miR-15a	0.0000176246	0.0002582749	Up	22.61393059
hsa-miR-18a	0.0000045344	0.0000220907	Up	5.974504433
hsa-miR-27b	0.0001433345	0.0217867113	Up	6.007352154
hsa-miR-28-3p	0.0000363064	0.0001763386	Up	24.12986015
hsa-miR-28	0.0000356773	0.0007026103	Up	12.20077435
hsa-miR-29b	0.0000045110	0.0000446240	Up	6.058264764
hsa-miR-29c	0.0000022294	0.0000109384	Up	10.6702369
hsa-miR-31	0.0000022218	0.0000152100	Up	20.82046385
hsa-miR-92a	0.0001413414	0.0014162922	Up	14.68638018
mmu-miR-93	0.0000088895	0.0001243419	Up	6.011333249
hsa-miR-99a	0.0000359286	0.0001740999	Up	7.386481816
hsa-miR-99b	0.0000699830	0.0004923074	Up	24.24865292
hsa-miR-100	0.0000047146	0.0001007295	Up	6.025744617
hsa-miR-101	0.0000713869	0.0003512175	Up	21.61277146
hsa-miR-106b	0.0000044494	0.0000598508	Up	11.79241078
hsa-miR-125b	0.0000363742	0.0003481664	Up	10.49672038
hsa-miR-130a	0.0000734506	0.0004906931	Up	29.05240133
hsa-miR-136	0.0000021992	0.0000609989	Up	10.89325593
mmu-miR-140	0.0000010873	0.0000076960	Up	5.975879236
hsa-miR-143	0.0000359259	0.0001759426	Up	23.88130556
mmu-miR-187	0.0002912525	0.0056576959	Up	9.763740353
hsa-miR-193a-3p	0.0000393223	0.0002445030	Up	2.254945919
hsa-miR-193b	0.0000023824	0.0000053720	Up	11.45048471
hsa-miR-196b	0.0002979137	0.0028274519	Up	20.31581999
hsa-miR-200a	0.0000022279	0.0000219849	Up	20.52730198
hsa-miR-210	0.0000371109	0.0004927418	Up	29.63419938
hsa-miR-218	0.0001432138	0.0040121562	Up	10.84716204
hsa-miR-339-3p	0.0000179084	0.0001242423	Up	11.86043563
hsa-miR-155	0.0001468812	0.0014070302	Up	11.77170329
hsa-let-7b	0.0001386657	0.0006844583	Up	6.118243605
hsa-miR-452	0.0000088236	0.0001757150	Up	24.59062971
hsa-miR-493	0.0000009831	0.0000109190	Up	11.11498543
hsa-miR-500	0.0000023069	0.0000154820	Up	10.41188492
hsa-miR-539	0.0000022609	0.0000153559	Up	10.39296695
hsa-miR-652	0.0000011157	0.0000306479	Up	19.51122157
hsa-miR-708	0.0000022561	0.0000213276	Up	10.27393174
hsa-miR-30a-5p	0.0000187059	0.0001242389	Up	18.52588049
hsa-miR-622	0.0000034524	0.0000211653	Up	6.211150264
hsa-miR-572	0.0000031769	0.0000409741	Up	13.08208849
hsa-miR-770-5p	0.0000127369	0.0001143333	Up	10.29941178
hsa-miR-99a	0.0000032457	0.0000142938	Up	5.045521643
hsa-miR-145	0.0000016463	0.0000141523	Up	8.73420763
hsa-miR-27b	0.0000064869	0.0000809041	Up	12.68090337
hsa-miR-378	0.0002220957	0.0006670456	Up	3.050953829
hsa-miR-151-3p	0.0000510329	0.0006649765	Up	18.30361098
hsa-miR-214	0.0000033395	0.0000575783	Up	19.56162482
hsa-miR-425	0.0000062023	0.0000410135	Up	6.704588903
hsa-miR-10b	0.0000068262	0.0000578115	Up	9.649507561
hsa-miR-181c	0.0000259252	0.0001144539	Up	5.042527682
hsa-miR-27a	0.0000031041	0.0000812814	Up	26.62109487
hsa-miR-1254	0.0000067522	0.0000561043	Up	9.44394700
hsa-miR-30b	0.0000716277	0.0000076840	Down	0.11270600
hsa-miR-223	0.0092950671	0.0001796576	Down	0.02408100
hsa-miR-454	0.0002824790	0.0000220106	Down	0.16349700
hsa-miR-486	0.0001542431	0.0000156434	Down	0.15936700
hsa-miR-574-3p	0.0005999424	0.0000857490	Down	0.17865900
hsa-miR-126	0.0000509905	0.0000070536	Down	0.15685000
hsa-miR-34a	0.0000064237	0.0000024902	Down	0.39429700
hsa-miR-625	0.0000135014	0.0000050833	Down	0.38285000
hsa-miR-1290	0.0000132930	0.0000052090	Down	0.39754900

miRNA, microRNA.
